# Unskilled Labor

**Published:** 1863-02

**Authors:** 


### UNSKILLED LABOR.

It would be a grand thing for society, if the apprenticeship system of fifty years ago could be resuscitated with modifications. A law would have its advantages, which would prohibit mechanics from setting up shop for themselves until a certain number of years had been spent in learning a trade. The want of something of the sort is diminishing daily the number of competent mechanics in every branch of human labor which requires intelligence and skill. Its working is as follows: A boy goes to "learn a trade." About the time it is half done, he begins to feel as if he knew all about it, and with that there comes a pride—a groundless independence and confidence in himself; the next step is to take offence at some trifling thing, and he "goes off," to become "Boss" himself. His next plan is, by "low prices," to get custom; but he would soon starve, if, with these low prices, he did not purchase a correspondingly inferior article to work with; and, with his incomplete knowledge, added to bad materials, "a bad job is made of it." People soon find him out, and simply let him alone; and he is forced to do anything that offers, make mortar, sweep the streets, saw wood, take up the hod, dig ditches, and the like. But such occupations are very precarious. There is not always work to do. There are rainy days, and snowy days, and days of frost; but he and his family must eat on these days as well as others, and he either goes in debt, or endeavors to live by his wits. He engages in unlawful practices, or associates with idlers, or hangs around drinking places, and the station-house, the jail, the penitentiary, and the gallows, close his history.

What is the remedy? Law? Do not wait for a law. Let every father who reads this, determine at once to use all the authority he has, if he designs to make a mechanic of his son, in compelling that son to become as much a master of his calling, as can possibly be done by the time he arrives at the age of twenty-one years; and, if not perfect in his business then, pay him as liberal wages as circumstances will allow, to induce him to remain until he is master of his calling; the result will be, that employers will feel a greater confidence that work will be well done, and with this will come higher wages, a more liberal remuneration, and more work; for many a man is prevented from making improvements, or having repairs done, because of the almost utter impossibility of having them done honestly and well. Thus it is that the great mass of mechanics are doomed to poverty for life; are doomed to live from hand to mouth; must live on each day's labor, and, without that labor, must stint, or cheat, or hunger. On the other hand, go to any first-rate workman in any branch, any day in the year, in a large city like New York, and you will never fail to find him "forehanded;" he always has work to do, and you are compelled to "wait your turn." First-rate mechanics are always in demand, and seldom fail, not only to make money, but to save it; and with that, to elevate themselves, their families, and their calling; but these are in such a minority, that the more numerous incompetent, and hence thriftless, class, are the ones who give character to the name of mechanic, which is too often a low one; when, if every one was a master workman, it would be but another name for industry, elevation, and thrift.

